# Mobile Schwannoma of the Lumbar Spine: A Case Report and Review of the Literature

**DOI:** 10.7759/cureus.715

**Published:** 2016-07-27

**Authors:** Daniel T Toscano, Daniel R Felbaum, Joshua E Ryan, Anousheh Sayah, Mani N Nair

**Affiliations:** 1 Neurosurgery, Medstar Georgetown University Hospital; 2 Neuroradiology, Medstar Georgetown University Hospital

**Keywords:** lumbar radiculopathy, schwannoma, ultrasound, neurosurgical complications

## Abstract

Mobile schwannomas of the spine have been sparsely documented in the literature. In cases referred to in existing literature, the migratory schwannoma was documented to occur in the lumbar spine. We added another case to the small available literature. In our case report, the patient had a previously known lumbar schwannoma that was being managed conservatively. Due to an acute change in clinical symptoms, repeat imaging was performed. A magnetic resonance imaging (MRI) of his spine revealed migration of the schwannoma two levels rostral to his recent imaging from six weeks earlier. The patient underwent surgical resection of his lesion. During the operation, the ultrasound was utilized to confirm the lesion prior to dural opening. In this report, we attempt to provide further evidence of the utility of an intraoperative ultrasound for intradural lesions and intend to add to the published literature of mobile schwannomas of the spine

## Introduction

Although rare, mobile schwannomas have been observed at various vertebral levels. Most of them have been reported within the lumbar spine. As described in the available literature, preoperative imaging confirmed the level of the operative intradural lesion. In several instances, imaging was performed just several hours prior to the operation. In a surgical nightmare, operative findings revealed normal spinal anatomy at the previously determined position [1-4]. Regarding these cases, subsequent exploratory laminectomy and extension of dural opening were not always successful in locating the lesion. When used for an intraoperative imaging, the ultrasound may help prevent an unsuccessful operation by providing real-time confirmation of the intradural location just prior to dural opening [3,5]. We present the case of a migratory lumbar schwannoma and the utility of an intraoperative ultrasound in confirming its location in aiding surgical resection.

## Case presentation

A 40-year-old gentleman presented with a history of progressive lower back pain and circumferential right leg pain. Over a period of one week, the pain developed into higher low back pain along with bilateral lower extremity weakness and increasing difficulty with balance. Previously, a magnetic resonance imaging (MRI) of his thoracolumbar spine demonstrated an intradural, extramedullary mass in the T11 to T12 intradural region.

**Figure 1:**
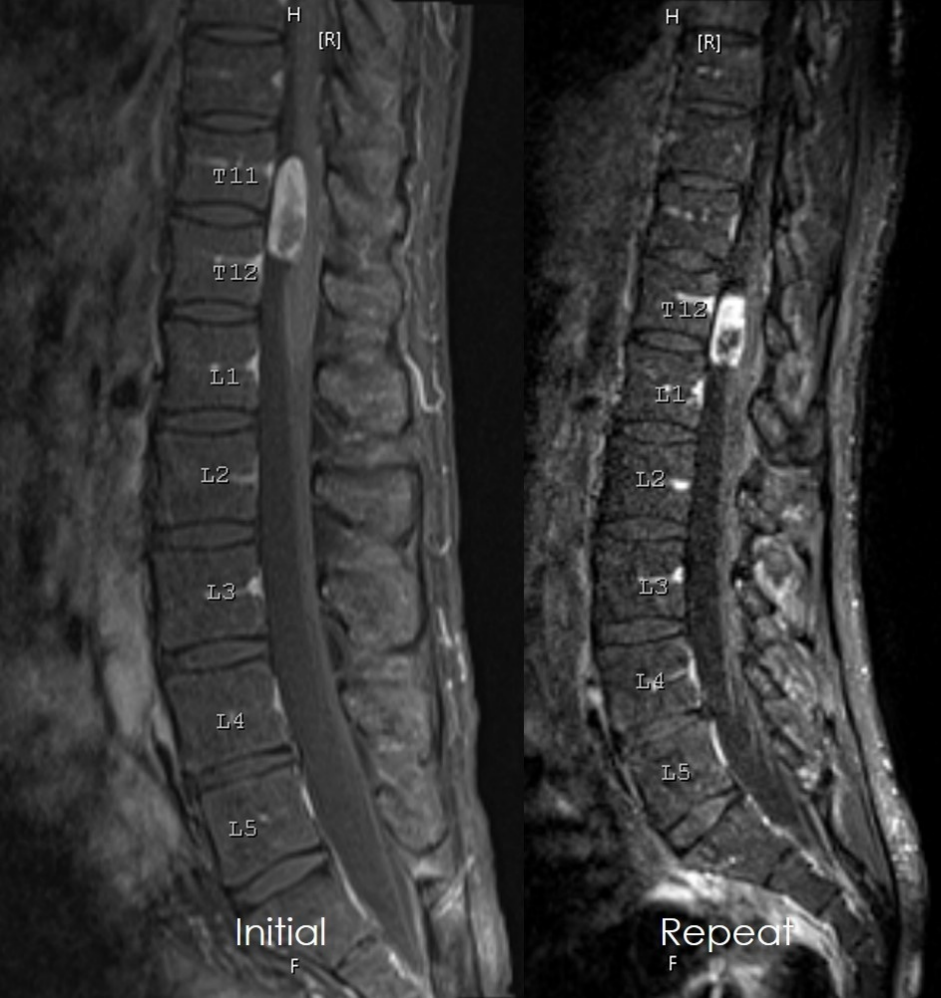
Initial and preoperative MRI imaging Initial sagittal MRI with gadolinium demonstrated an intradural extramedullary mass in the T11-T12 region. Immediate preoperative imaging obtained after new onset bladder incontinence demonstrated tumor movement caudally, down to the T12-L1 region.

A repeat MRI was performed due to the new onset bladder incontinence. Upon further review of his most recent imaging, the lesion was located at T12-L1, no longer as high as T11 (Figure 1).

**Figure 2:**
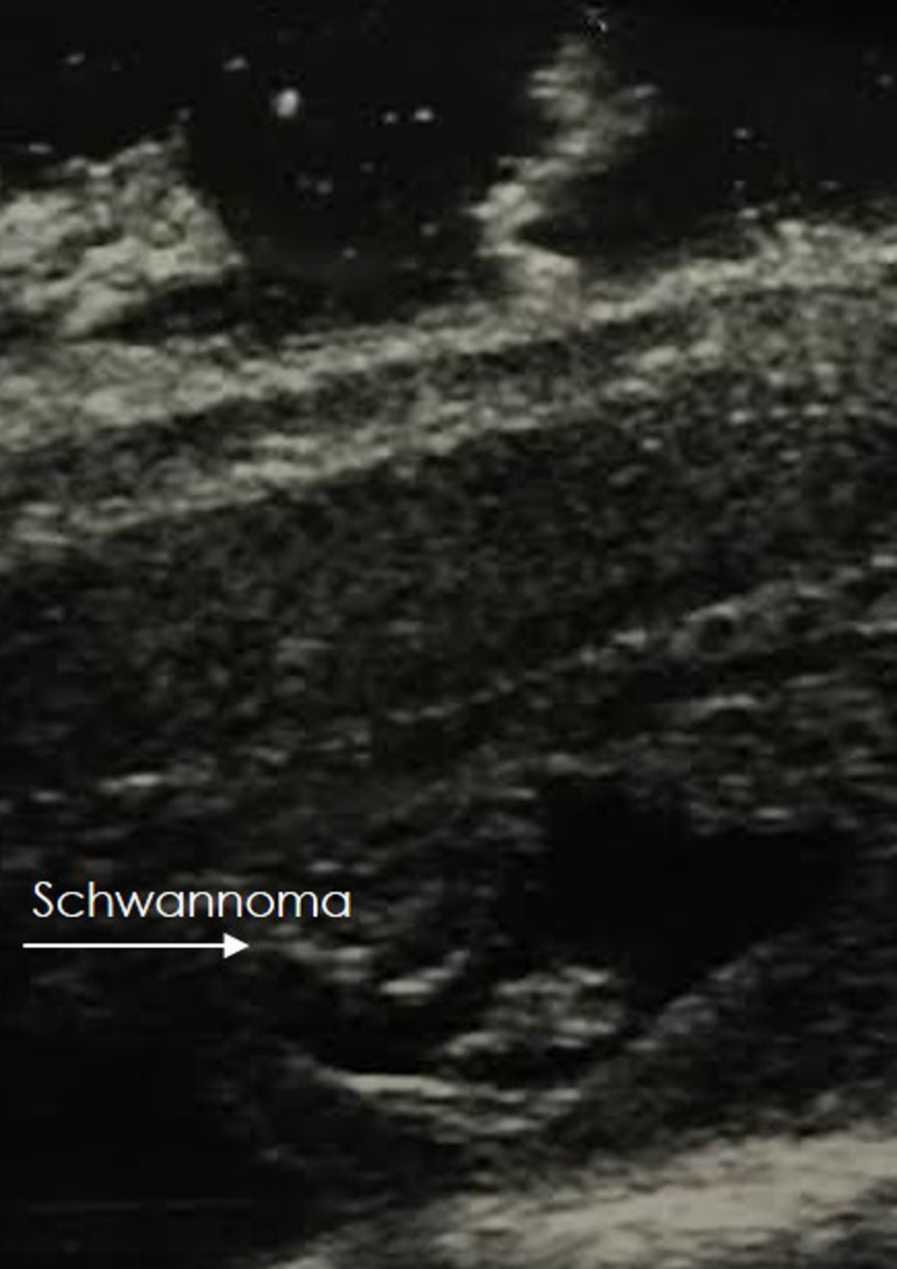
Intraoperative ultrasound An intraoperative ultrasound confirmed the location of the intradural lesion just prior to the opening of the dura.

Informed consent was acquired for the study. The patient was then taken to the operating room for surgical excision of the lesion. An intraoperative ultrasound confirmed the intradural location and the resection went without untoward events (Figure 2).

Pathology was consistent with a schwannoma. Ultimately, the patient had an unremarkable hospital stay and was discharged to an acute rehabilitation facility.

## Discussion

Mobile schwannomas of the spine are rare with only 18 computer tomography (CT)/MRI confirmed cases reported to date, this one included. Table 1 displays all reported cases of mobile spinal schwannomas diagnosed using CT and MRI [1-10].

**Table 1:** Summary of Published Cases of Mobile Spinal Schwannomas Summary of availalbe literature regarding mobile schwannomas of the spine

Author/Year	Age/Sex	Initial Location	Final Location	Discrepancy (Vertebrae)	Migration	Imaging Modality	Additional durotomy and/or lengthening of original incision?
Isu et al [6] 1989	51F	T11-T12	T12-L1	1	Caudal	MRI	Unknown
	42M	T12	L1	1	Caudal	MRI	Unknown
	52M	L1	L1-L2	1/2	Caudal	MRI	Unknown
Namura et al [7] 1993	51M	T4-T5	T9-T10	5	Caudal	Myelography, MRI	No
Varughese et al [2] 1997	65M	L5	L4	1	Rostral	Myelography, CT	Unknown
	78M	L3	L2	1	Rostral	Myelography, CT	Unknown
Iizuka et al [8] 1998	48M	C7-T1	T1-T2	1	Caudal	MRI, intraop-Myelography	Unknown
Friedman et al [9] 2003	28M	L4	L3	1	Rostral	MRI, intraop-US	No
	33M	L5	L4-L5	1/2	Rostral	MRI, intraop-US	No
	41F	L2	L1-L2	1/2	Rostral	MRI, intraop-US	No
Marin-Sanabria et al [3] 2007	27M	L1	L1-L2	1/2	Caudal	MRI, intraop-MRI	Yes, L1 $\rightarrow$ T12-L2; L2*
	41M	L2-L3	L1-L2	1	Rostral	MRI, intraop-MRI	Yes, L2-L3 $\rightarrow$ L2-L4; L1-L2*
Kim et al [4] 2009	45M	L3-L4	L2-L3	1	Rostral	MRI	No
	32M	T10-T11	T11	1/2	Rostral	MRI	Yes, T10 $\rightarrow$ T10-T11
	27M	L3-L4	L2-L3	1	Rostral	MRI	Yes, L3-L4; L2*
Khan et al [1] 2013	52M	T10-T11	T7-T8	3	Rostral	MRI	Yes, T9-T11 $\rightarrow$ T9-T12; T7-T12*
Terada et al [10] 2016	68M	C5-C7	C6-T1	1	Caudal	MRI, intraop-US	No
Toscano et al, 2016	40M	T11-T12	T12-L1	1	Caudal	MRI, intraop-US	No
*Second Surgery							

When comparing preoperative tumor location and final tumor position upon excision, each case demonstrates a significant change in the vertebral level of the lesion. Primary factors driving tumor movement include postural adjustments and arrangement on the operating table as well as positive pressure respiration and other actions that influence intrathecal, intrathoracic, or intra-abdominal pressures such as the Valsalva maneuver [2, 3, 4, 7, 9-10]. Surgical and imaging procedures such as laminectomy and contrast injection also affect tumor position [3]. Also, tumors originating in the cauda equina may exhibit heightened positional freedom due to an increase in lumbar nerve root length and the absence of a solid cord structure which is present in cervical and thoracic schwannomas [4, 10]. In our case, this may have contributed to the lesion’s mobility observed on imaging.

The enhanced risk of surgically removing a mobile schwannoma comes not from dural attachment as almost all cases reported no dural adhesion. Instead, they arose from an unpredictable and often substantial movement of these lesions up until the moment of excision [4, 9]. With their patient post L1-L3 laminectomy and just prior to dural opening, Varughese et al. reported observing a mobile schwannoma oscillating between L1 and L3 in step with each respiration [2]. As dural incision is necessary to remove intradural mobile schwannomas, it is important to confirm tumor location intraoperatively before entering the subdural space as the tumor may have shifted since preoperative imaging. Several cases reported finding no lesion after initial dural incision at the preoperatively determined location thus necessitating further exploratory laminectomy and durotomy [3, 4]. An intraoperative myelography has in the past, and can be used to verify tumor-positon before durotomy. However, the injection of a contrast material may itself induce tumor mobility [4]. In two separate cases, Marin-Sanabria et al. reported the use of an intraoperative MR imaging to determine tumor location after initial laminectomy through the dural opening was unsuccessful. It is also worth noting that access to intraoperative MR may not be feasible in many operating suites [3].

An intraoperative ultrasound has proven very effective in tumor localization before entering the dural space and does not significantly increase surgical risk or procedure length [9]. Additionally, an intraoperative ultrasound has inherent benefits over an intraoperative myelography and an MR imaging. The reason being, the ultrasound procedure does not introduce variations of intrathecal pressure, and anatomical changes can be observed in real time. In the event of tumor mobility, the ultrasound may help to determine precisely the levels of lamina that need to be removed. When considering the cases where the ultrasound was used, this technique is successful in confirming or appropriately modifying the site of the dural incision. As observed in our particular case, schwannomas involving the cauda equina may have more variability and potential to migrate. An intraoperative ultrasound was essential in minimizing morbidity by confirming the location of the lesion prior to dural opening. In further operations involving intradural lesions, schwannomas of the cauda equina in particular, an intraoperative ultrasound should be utilized.

## Conclusions

Migratory schwannomas of the spine are rarely reported in the literature. We add to the available literature another case of a mobile lumbar schwannoma. Due to its location at the cauda equina, a schwannoma in this region may be more prone to migration. An intraoperative ultrasound is essential in confirming operative location prior to dural opening. Also, we present a brief review of the available literature regarding this unusual phenomenon.
